# Embedded Strain Gauges for Condition Monitoring of Silicone Gaskets

**DOI:** 10.3390/s140712387

**Published:** 2014-07-10

**Authors:** Timo Schotzko, Walter Lang

**Affiliations:** Institute for Microsensors, Actuators and Systems (IMSAS), University of Bremen, Otto-Hahn-Allee NW1, 28359 Bremen, Germany; E-Mail: tschotzko@imsas.uni-bremen.de

**Keywords:** strain gauge foil, smart gasket, embedded sensor, structural health monitoring, silicone O-rings, miniaturized sensor

## Abstract

A miniaturized strain gauge with a thickness of 5 µm is molded into a silicone O-ring. This is a first step toward embedding sensors in gaskets for structural health monitoring. The signal of the integrated sensor exhibits a linear correlation with the contact pressure of the O-ring. This affords the opportunity to monitor the gasket condition during installation. Thus, damages caused by faulty assembly can be detected instantly, and early failures, with their associated consequences, can be prevented. Through the embedded strain gauge, the contact pressure applied to the gasket can be directly measured. Excessive pressure and incorrect positioning of the gasket can cause structural damage to the material of the gasket, which can lead to an early outage. A platinum strain gauge is fabricated on a thin polyimide layer and is contacted through gold connections. The measured resistance pressure response exhibits hysteresis for the first few strain cycles, followed by a linear behavior. The short-term impact of the embedded sensor on the stability of the gasket is investigated. Pull-tests with O-rings and test specimens have indicated that the integration of the miniaturized sensors has no negative impact on the stability in the short term.

## 1. Introduction

Rubber and silicone gaskets are usually small yet crucial components in many technical systems. A gasket is usually a low-cost product and comprises only a small part of the overall cost of the total system. However, its role in safety and long-term function is significant. Defective seals can sometimes result in disproportionate failures; a classic example is the explosion of the Space Shuttle Challenger in 1986, which was caused by the failure of a rubber O-ring [1]. At present, it is impossible to directly monitor the condition of the gasket in its in-service state; hence, reaction to a possible failure or leakage cannot be performed preemptively. Consequently, gaskets must be periodically replaced to avoid failures, especially in security-critical applications. This schedule-based rather than needs-based approach leads to extra effort and expense. One possibility for monitoring the state of an O-ring is to embed sensors into the gasket.

Various factors can affect the functionality of rubber gaskets. In addition to ordinary aging of the materials, degradation can be hastened by contact with incompatible chemicals and gases and by unsuitable temperatures. The elastomeric materials become porous and a transition from elastic to viscoelastic behavior occurs. As a consequence, the compression set increases, and the gasket retains its shape even after pressure has been released. The reset force of the gasket decreases and leakage occurs. Another reason for a failure may be from an incorrect assembly or fitting. A faulty gasket and uneven or excessive compression often lead to malfunctioning. On the other hand, insufficient compression can directly lead to leakages [2].

O-rings are found in many applications. Depending on usage, a defective gasket can present a high safety risk or danger to the environment, e.g., in areas where hazardous chemicals and gases are sealed. A malfunction of these low-cost components can also produce disproportionately high costs. Many rubber gaskets, for example, are built into pneumatic and hydraulic systems, and leakages affect the energy efficiency of such systems. A study from 2001 about “compressed air systems in the European Union” has shown that high energy losses occur due to lack of preventive maintenance. The study assumes that prevention of air leaks could save up to 12.8 billion kWh a year in the European Union (in 2001). This corresponds to 16% of the total energy consumption in this field [3]. Therefore, monitoring the condition of single gaskets is of great interest because the occurrence of leakages could be diminished and the need for extensive preventive maintenance could be avoided.

As an example for a self-monitoring gasket, this study presents the design and performance of an embedded strain gauge for O-ring monitoring. Through this technology, the deformation of a gasket can be measured, and the contact pressure determined. The integration of a strain gauge could provide a promising basis to embed additional sensors in silicone and rubber materials, including temperature sensors and sensors to measure dielectric permittivity.

### 1.1. Structural Health Monitoring

Sensor integration has become an important research topic over the past decade. Structural health monitoring (SHM) is a highly discussed topic and continues to attract interest in different fields. The areas of application have been enlarged in recent years, and systems have become increasingly complex. Since the late 1980s, this idea has been established in the fields of aerospace, civil, and mechanical engineering [4–6]. The aim of SHM is to provide a continuous diagnosis of the state of the controlled material and structure in a nondestructive manner. Thus, defective materials, damage from external influences, and aging processes can be monitored and evaluated. The results can be used to provide a diagnosis of the development of damage and a prognosis of the remaining service life [7]. Embedding sensors in O-rings, as a part of SHM systems, is a new approach of great interest to many industrial applications.

The integration of sensors presents two major challenges. On the one hand, the sensors should be designed such that the host material will not be damaged, although any embedded structure always represents an interruption to the material. On the other hand, it is also important that the sensor provides a reliable, repeatable, and preferably linear response to the parameters being measured.

**Figure 1.**
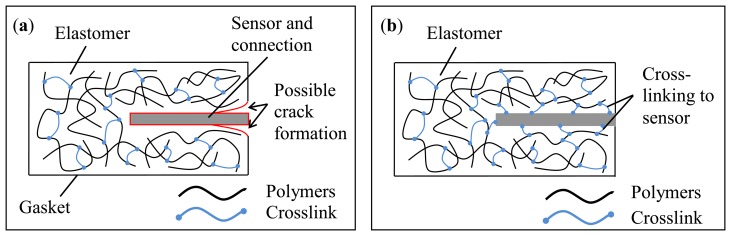
A sketch of an embedded sensor in a gasket (**a**) with no crosslinking agents between sensor and elastomer, which exhibits a possible source of crack formation and separation; (**b**) with crosslinking between host material and sensor structures.

Therefore, the sensors must be designed with a size and choice of material such that it does not weaken the structure of the gasket, but still provides the information needed to monitor the condition [8]. One possibility to minimize the influence of the embedded sensor is to select a material for the sensor substrate, which is chemically similar to the host material. Thus, it is possible to enable crosslinking between sensor and gasket, as shown in Figure 1.

### 1.2. State of the Art

#### 1.2.1. Sensor Integration

Sensor integration together with SHM is a generally topical subject. The data collected by integrated sensors can provide various types of information about the host material. The evaluation of the received data is of great interest for research and development. It can reveal the behavior and changing properties of a host material and a hosting device online. This allows us to gain greater comprehension of materials themselves and of the whole system. It also enables several new possibilities of analysis for monitoring during implementation as well as testing the properties of materials and devices. For gaskets, it could also complement standard rubber testing methods with applicable sensors. For example, it would be possible to measure the chemical change in the rubber material during exposure to harmful substances. Through the embedded nature of the sensors, a more precise evaluation of the impact characteristics of the material would be possible.

In recent years, different sensors have been successfully integrated in various materials, such as metals [9–11], textiles [12,13], and fiber-reinforced polymers (FRPs) [5]. Temperature sensors have been embedded in aluminum alloys to detect temperatures during molding processes [9]. Other groups have integrated thermoelectric energy generators [10] and a radio-frequency identification transponder [11] in aluminum by casting. The integration of sensors in metals is highly challenging because of the harsh conditions during metal casting, rolling, and forming processes.

In contrast to the challenges associated with embedding sensors in structural materials are those associated with embedding sensors into textiles. These materials are usually very flexible and stretchable. Therefore, the sensors have to be compliant and operate over wide strains. They must also be resistant to chemicals, soaps, and perspiration. The embedding processes are different in this case as well. Instead of molding, the sensors must be woven in the materials. Common usage of integrated sensors and electronics is in smart textiles for sports, lifestyle, and the health industry. Clothing that includes sensors for monitoring an electrocardiogram [12] and shoes with energy-harvesting microelectronics have already been demonstrated [13], among many other examples.

FRPs and carbon-fiber-reinforced polymers (CFRPs) are commonly used as rugged and lightweight materials in aircrafts and in wind turbine blades. Possible applications for integrated sensors are measuring acting forces, monitoring fiber breakage, and observing the change in permittivity at the interface between the sensor and the host material, caused by polymerization within the manufacturing process. To avoid delamination, the sensors have to be miniaturized as well as flexible. Integration into the CFRP of a flexible sensor based on polyimide, with a thickness of about 5 µm, has already been demonstrated [14].

#### 1.2.2. Common Gasket Leakage Detection Methods

Leakages are commonly detected by indirect methods. Different pressure and flow sensors are usually integrated in industrial facilities to indirectly monitor the symptoms of leakage rather than directly monitor early gasket failure. Thus, a loss in process pressure and vacuum can be detected, and a leakage rate can be calculated. A significant disadvantage of this method is that the exact location of the defective gasket is not directly indicated [15,16]. Therefore, these types of monitoring approaches are rarely implemented in industrial facilities. The most prevalent method for directly finding a defective gasket is performed manually using an ultrasound detector. The detector can identify the ultrasound emission of a leak, thus enabling localization of the leakage at the site of the faulty gasket [17].

A different approach for detecting faulty gaskets is through external gas sensors, usually applied in chemical process plants. In an emergency, if poisonous or inflammable gas leaks, the plant can be shut down to find the leakage. In addition, other methods exist to detect leakage of liquids through defective gaskets. Seals with an interior repository can collect leaking liquids, such as oil, that are detectable [18]. However, all the above mentioned leakage-detection methods require significant installation effort, are bulky, and are associated with high expense.

#### 1.2.3. Gaskets with Sensors

Gaskets with sensors have been investigated for considerable time and via many different methods, some of which have proved successful. One of the most widely known is the radial shaft seal with an integrated leakage sensor, which is currently used in wind turbines [18]. These gaskets detect leaked liquids (normally oils) and are usually made of an interconnected system of metal and rubber. The condition of the gasket is indirectly monitored by detecting oil in a depot inside the gasket using an optical sensor. The range of applications is limited for this type of gasket. Other examples are cylinder head gaskets with integrated temperature sensors, which enable optimized engine management [19] as well as a plastic gasket with a sensor array on it. The sensors detect fault assembling, leakages, and chemical and physical aging processes of the gaskets [20].

### 1.3. Concept of Condition Monitoring for Gaskets by Using Strain Gauges

**Figure 2.**
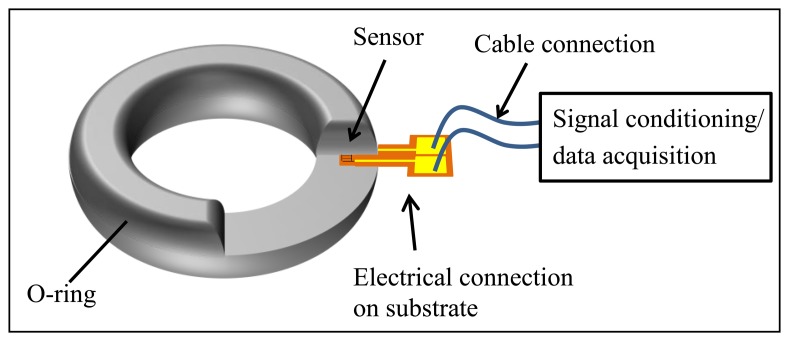
Illustration of an embedded strain gauge in a silicone gasket.

The motivation of the current study is to obtain as much information as possible about the condition of a gasket through embedded sensors. Various parameters, such as compression set, permittivity, and contact pressure, could provide relevant information. In this article, a strain gauge is described as a gauge that measures the contact pressure of the gasket. The concept is to integrate a strain gauge centered within an O-ring, so that radial strain can be detected by the sensor. The strain arises from vertical pressure applied to the gasket. The embedded strain gauge is elongated under the gasket contact pressure, which leads to a measurable increase in resistance. The connection is made with gold conducting paths on the same thin and flexible substrate as the sensor. These lead outside the gasket, so that they can be connected to a measurement unit. Thereby, the resistances of the contact paths are much lower than that of the strain gauge structure to minimize loading of the measurement signal. The structure of the embedded sensor in the gasket and the connections are shown in Figure 2.

## 2. Experimental Section

**Figure 3.**
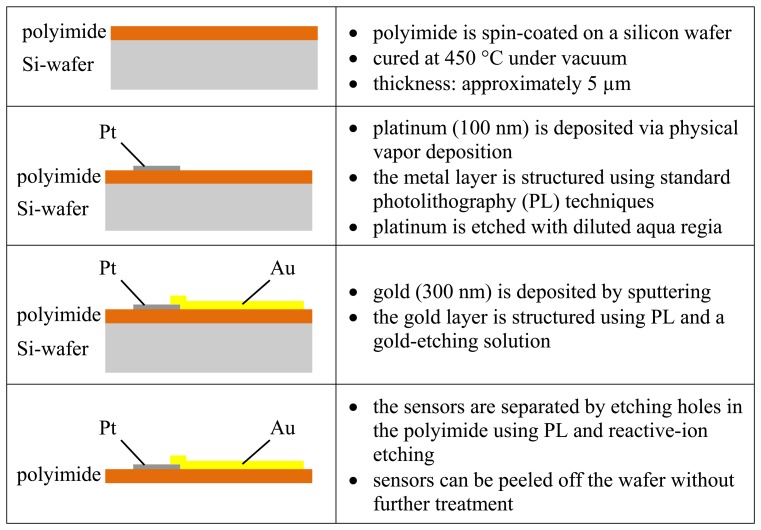
Summary of the processing steps of the sensor.

The strain gauges are processed as a thin-film platinum resistor on polyimide. A variety of different meander structures are implemented. Gold is used for interconnections on the polyimide foils. Subsequently, the sensors are molded into silicone O-rings and examined. An overview of the single- processing steps of the sensors is presented in Figure 3.

**Figure 4.**
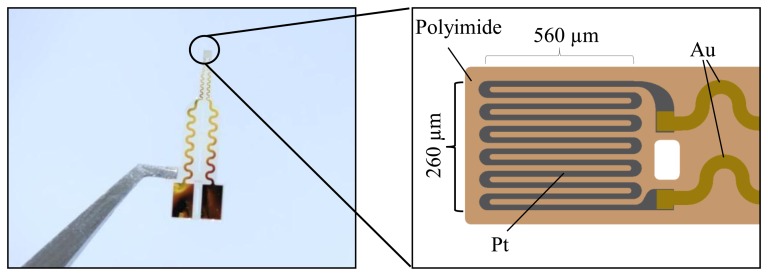
Photograph of a strain gauge and schematic of the meander structure.

A 5-µm-thick polyimide layer is spin-coated on a silicon wafer and annealed under vacuum. Platinum is deposited on the polyimide and structured by standard photolithography (PL) techniques and diluted aqua regia. In addition, the gold conductive paths and pads are sputtered on and patterned by PL. The single strain gauges are separated by etching the polyimide foil using reactive-ion etching. Because of the annealing process of the polyimide on silicon, the sensors (see Figure 4) can be simply peeled off from the silicon wafer with no further treatment.

**Figure 5.**
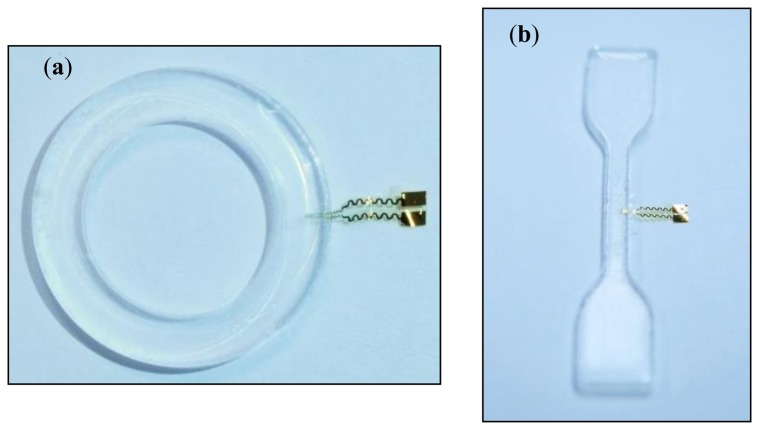
Photographs of an embedded sensor in (**a**) an O-ring and (**b**) a test specimen.

In the second step, the gaskets and test specimens are fabricated. For this, a casting mold with a bracket for the sensors is used to embed the strain gauges. The gauge pattern is located inside the casting mold when liquid silicone is molded. One strain gauge is embedded in each O-ring and each test specimen, as shown in Figure 5. The silicone gasket is cured for 20 min at 120 °C.

### 2.1. Sensor Validation and Performance Characterization

#### 2.1.1. Validation of Embedded Sensor

**Figure 6.**
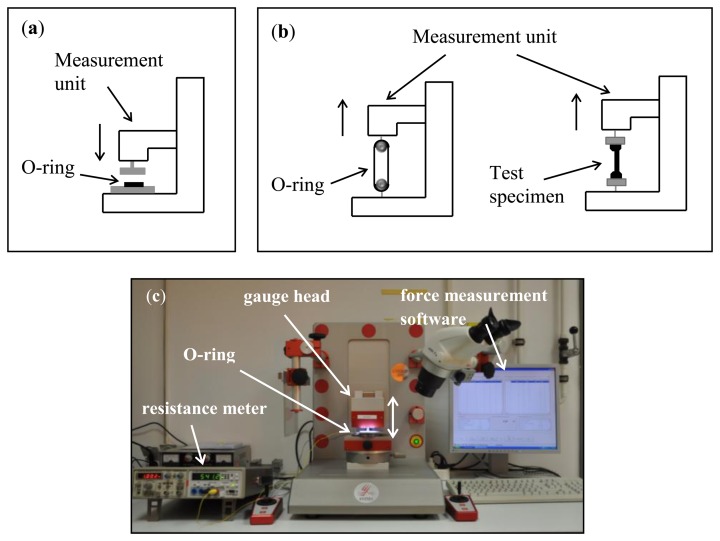
Sketches of the measurement setup. (**a**) for pressure tests; (**b**) pull-tests; (**c**) photograph of the measurement setup for pressure test.

The prepared gaskets are placed between two metal plates in a force-measurement unit (XYZTEC Condor with pull-and-push measurement unit) as shown in Figure 6a,c. The sensors are connected by two cables to a resistance meter (HM8012 programmable multimeter) while the gauge head applies pressure on the O-rings. The measurement head is moved in 100 µm steps in a range of 0–1500 µm. The force of the applied pressure is monitored and correlated with the resistance of the strain gauge.

#### 2.1.2. Uniaxial Tensile Testing on Dog-Bone Test Specimens

With the same measurement unit, we also perform tensile testing (Figure 6b). Therefore, dog-bone test specimens are produced with and without embedded sensors. The strain gauges are centrally placed within the test specimens, as illustrated in Figure 5b. Subsequently, they are tested in uniaxial tensile to failure. The elongation of the specimens is recorded against the applied force. The fracture strengths of the dog-bone test specimens with and without embedded sensors are compared.

#### 2.1.3. Uniaxial Tensile Testing on O-rings

Uniaxial tensile testing is also performed on the O-rings using suitable hooks (Figure 6b). In this case, the fracture patterns of the O-rings with integrated sensors are evaluated to determine whether the sensor affects the point of fracture compared with O-rings without sensors. On the basis of the tensile testing, a statement can be made about a possible influence of the embedded sensors on the performance stability of the gasket.

## 3. Results and Discussion

The produced strain gauges have resistances between 600 and 3300 Ω because of the different length, width, height, and distance of the strain gauge patterns. Currently, no evidence exists that the curing process during the embedding into silicone has any influence on the sensor resistance.

### 3.1. Force-Resistance Measurements

**Figure 7.**
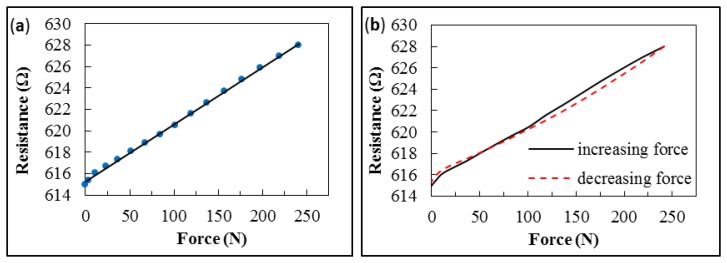
Resistance–force characteristic of a strain gauge embedded in a silicone O-ring. (**a**) Nearly linear response while increasing force; (**b**) Hysteresis during the first strain cycle.

The force–resistance response of the embedded sensor exhibits linear behavior at a constant temperature (room temperature), as illustrated in Figure 7a.

At low forces (<20 N), the graph shows a slight deviation, caused by the surface characteristics of the O-rings. As a result of the production process, the gasket is slightly thicker around the embedded sensor. Hence, the O-ring surface touches the measurement unit unevenly. The contact pressure is not applied uniformly across the O-ring at the beginning of the measurement. This leads to an irregular strain distribution that causes the observed deviation in resistance of a strictly linear response. However, as soon as the metal plate is consistently pressed onto the gasket (usually at about 20 N contact force), the measured data exhibits a proportional relationship between contact force and resistance.

**Figure 8.**
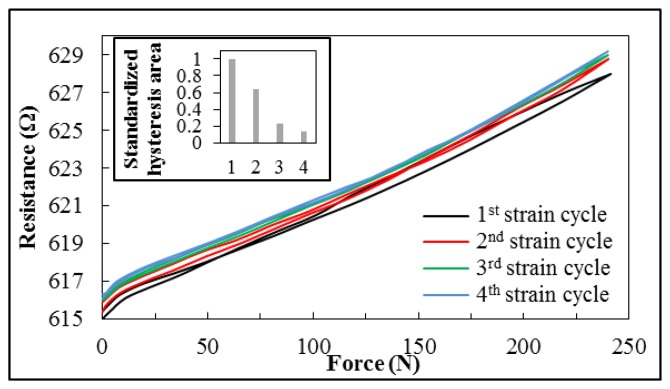
Hysteresis effect of resistance–force response during first four cycles of loading and unloading the strain gauge. The bar chart points out the change in area of the hysteresis loops.

Although the strain gauge exhibits linear behavior in response to increased applied force, a hysteresis effect was observed during the removal of force from the gasket (see Figure 7b). At high contact forces, the measured resistance of the strain gauge is lower when force is being reduced. However, with further reduction in force, the decrease lessens, with the result that a zero point offset of the resistance occurs toward higher values after unloading the strain gauge. This performance can be observed during the first four cycles. The hysteresis effect decreases each time, until there is almost no difference in behavior during cycling of the force, as shown in Figure 8. The zero offset is also negligible.

Hysteresis effects are already well described for strain gauges. These effects can be caused by cold hardening of the metals, properties of adhesion to the substrate material, or, in this case, the host material of the strain gauge foil causes hysteresis as well as the zero offset [21–23]. The hysteresis depends on the maximum applied strain and the number of cycles [21], as observed during these measurements. Another possible reason for the hysteresis is that during the curing process of the silicone, residual tensile strains are locked in the O-ring. Alternating strain and relaxation could relieve these residual strains and lead to a minor creep deformation of the silicone followed by a zero offset.

### 3.2. Uniaxial Tensile Testing

Preliminary results have shown that the integration of small sensors does not influence the stability of silicone O-rings. However, long-term measurements have not yet been performed. Accordingly, a negative influence over time through introduction of the sensors cannot be ruled out at this point.

Investigations to indicate whether the embedded strain gauges weaken the structural stability of the O-rings showed that no negative impact caused by the sensors could be observed. Tensile testing is performed on the gaskets and the test specimens, both with and without embedded sensors. The fracture strength is detected by the same measurement unit mentioned in Section 2.1.

#### 3.2.1. Test Specimens

**Figure 9.**
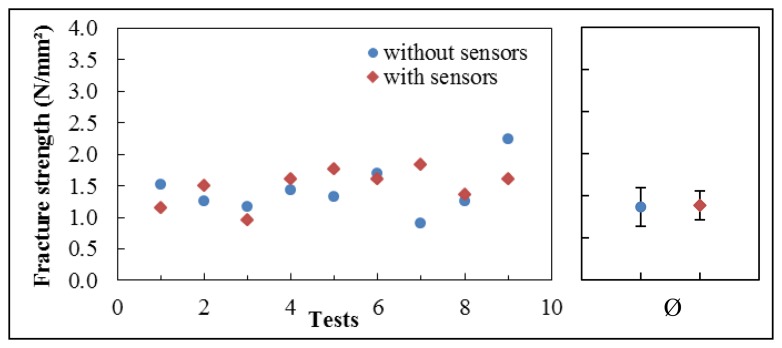
Comparison of the tensile testing results of the dog-bone test specimens with and without integrated sensor.

The results of the tensile testing show no differences between the dog-bone specimens with or without an embedded sensor. A range of measurement data is shown in Figure 9. The average fracture strength of all measurements is 1.44 N/mm^2^ (σ = 0.38 N/mm^2^) without sensors and 1.48 N/mm^2^ (σ = 0.29 N/mm^2^) with the embedded sensor.

#### 3.2.2. O-Rings

Uniaxial tensile testing with O-rings gives the same results. No differences are observed between silicone O-rings with or without an embedded sensor, neither by destructive forces nor by rupture location. The gaskets tear apart randomly and not specifically where the strain gauge is located. This shows that the sensors do not influence the integrity of silicone gaskets in the short term.

## 4. Conclusions and Outlook

A first approach in integrating strain gauges in silicone gaskets has been shown. The relationship between the contact force and the sensor resistance has been analyzed. A nearly linear behavior between the output signal and the force was observed, although hysteresis was observed during the first few cycles of contact-force application. Tensile testing has indicated that there are no negative effects on the stability of the gasket caused by the embedded sensors, at least in the short term.

These results are very promising for further research on self-monitoring gaskets. They provide the basis for integrating various sensors for condition monitoring of gaskets. Future work should be implemented to examine the long-term effects of embedded sensors on the host materials. Further, the sensor itself can be improved using different substrate materials to support an enhanced bond between the integrated structures and gasket material. In this context, other materials that can be linked through covalent bonds to the host material can be investigated to explore positive impacts on the stability of the gasket.

In future work, we will investigate methods to detect potential degradation of gaskets. Degradation changes the elastic behavior from elastic to viscoelastic, which in turn causes a loss of sealing pressure. It is expected that the change in elastic behavior is accompanied by deformations that are small but measurable by strain gauges. However, the experimental validation is yet to be performed and is a topic of future research work.
